# No strong support for a Dunning–Kruger effect in creativity: analyses of self-assessment in absolute and relative terms

**DOI:** 10.1038/s41598-024-61042-1

**Published:** 2024-05-24

**Authors:** Izabela Lebuda, Gabriela Hofer, Christian Rominger, Mathias Benedek

**Affiliations:** 1 Institute of Psychology, University of Graz, Universitätsplatz 2, 8010 Graz, Austria; 2 Institute of Psychology, University of Wrocław, Dawida 1, 50-527 Wrocław, Poland

**Keywords:** Human behaviour, Problem solving

## Abstract

Competencies related to the evaluation of own cognitive processes, called *metacognitive monitoring*, are crucial as they help decide whether to persist in or desist from cognitive efforts. One of the most well-known phenomena in this context—*the Dunning–Kruger effect*—is that less skilled people tend to overestimate their performance. This effect has been reported for various kinds of performance including creativity. More recently, however, it has been suggested that this phenomenon could be a statistical artifact caused by the *better-than-average* effect and by *regression toward the mean*. Therefore, we examined the *Dunning–Kruger effect* in the context of creative thinking performance (i.e., divergent thinking ability) across two studies (Study 1: *N* = 425; Study 2: *N* = 317) and applied the classical quartile-based analysis as well as newly recommended, advanced statistical approaches: the *Glejser test of heteroscedasticity* and *nonlinear quadratic regression*. We found that the results indeed depended on the employed statistical method: While classical analyses supported the Dunning–Kruger effect across all conditions, it was not consistently supported by the more advanced statistical methods. These findings are in line with recent work challenging certain assumptions of the Dunning–Kruger effect and we discuss factors that undermine accurate self-assessments, especially in the context of creative performance.

## Introduction

The accurate assessment of one’s cognitive performance and its effects—*metacognitive monitoring*—is one of the most critical metacognitive subcomponents^1^. Self-assessment of cognitive performance informs decision-making and goal-setting^2–5^. If this assessment is inaccurate, it could have severe consequences such as premature termination of the task engagement or unnecessary prolongation of ineffective performance approaches. Although there has been much interest in the accuracy of people assessing their own performance across many domains^5,6^, so far, there is only little work focusing on creativity. Creativity, understood as the mental activity that generates novel and useful ideas^7^, may not be so different from other complex cognitive skills^8,9^, yet some of its characteristics make self-assessment highly specific and demanding. Creative ideation relies on divergent thinking and thus is ill-defined, as there are infinite possible solutions and a lack of clear, objective assessment criteria for what is considered creative^10^. More ambiguous performances are more prone to uncertainty and biases in self-estimations due to an absence of direct-access cues or immediate feedback^11–13^. Therefore, it is crucial to take a closer look specifically at the relationship between self-assessment and actual performance in creativity.

Creative performance strongly relies on effective metacognitive processes such as metacognitive monitoring^14–16^. Creative people are thought to be *doubly skilled*: They do not only have more creative ideas but are also more discerning when judging the creative quality of their own and others' ideas^10,17–19^. However, there has been limited research on how people evaluate their overall creative performance (e.g., compared to others). Specifically, are less creative people also less accurate in their performance self-assessment, as suggested by the popular Dunning–Kruger effect^20^? Here we test the Dunning–Kruger effect in two empirical studies in the context of divergent thinking ability.

### Unskilled and unaware: assumptions and concerns about the Dunning–Kruger effect

The Dunning–Kruger effect, also called “unskilled and unaware” effect, is one of the best-known biases in metacognition. Research across different domains (e.g., logical reasoning, English grammar, and humor) indicated that people tend to overestimate their performance (*inflated self-assessment*), and that overestimations are more pronounced in those with the lowest skills^11,20^. The authors referred to it as a “double burden”, as having poor skills in a given domain also seemed to undermine an accurate self-assessment. In contrast, people who perform well tend to underestimate their skills. Kruger and Dunning^20^ argued that this miscalibration might not be due to a bias in assessing one’s own performance per se but rather result from the *false-consensus effect*—the incorrect assumption that others performed equally well as oneself (i.e., the overestimation of others' potential by creative people).

While Dunning–Kruger effects have been widely discussed across topics as diverse as health literacy or endorsement of anti-vaccine policies^21,22^, there have also been concerns about the underlying analysis approach. The most common method of testing the Dunning–Kruger effect, as proposed in the original paper, is to plot and compare the average self-assessed and objectively assessed skill across quartiles of performance^20^. However, many have argued that this method could lead to statistical artifacts due to two well established phenomena: the better-than-average effect and regression to the mean^23–26^ for a similar explanation see^27,28^. The better-than-average effect represents the tendency that most people, not only low-performers, judge their performance as above average^29,30^. Therefore, a quartile-based approach would necessarily lead to the highest miscalibration for the bottom quartile, as here the average performance is most different from above-average^23^. The second phenomenon of regression to the mean describes that in two imperfectly correlated variables (such as self-assessed and measured performance), relatively extreme values on one variable map to values closer to the mean on the second variable for purely statistical reasons^31,32^. Even though Kruger and Dunning^20^ acknowledged that regression to the mean may have impacted their results, they still attribute the Dunning–Kruger effect mostly to their proposed psychological mechanism (i.e., metacognitive inability among less skilled people). Nevertheless, simulation-based research suggests that regression to the mean alone^23^ or in combination with an above-average effect^33^ yield results that may be misinterpreted as the Dunning–Kruger effect—even in random variables.

To address this problem, Gignac and Zajenkowski^33^ proposed two new approaches to test the Dunning–Kruger effect. The first one, the Glejser test^34^, is a measure of heteroscedasticity. If low-performers evaluate their performance more inaccurately, their assessments should be associated with higher absolute residuals when regressed onto measured performance. Thus, a systematically higher heteroskedasticity for low performers as observed by a negative correlation between performance and the absolute self-assessment/performance-residuals would support the Dunning–Kruger effect. Another recommended test of the Dunning–Kruger effect is nonlinear (quadratic) regression, which tests the related assumption that that the correlation between self-assessed ability and measured ability should increase with higher performance levels. Here, the Dunning–Kruger effect would be confirmed if a positive monotonic quadratic effect is observed in the data. Both approaches have been recommended as they offer unique information about the relationship between self-assessed and objectively measured ability. Later research, however, indicated that the Glejser test may be a less direct test of the Dunning–Kruger effect as the absolute residuals do not allow for a distinction between over- and underestimation^35^. Specifically, a negative Glejser correlation indicates higher misestimation by low-performers but provides no information about the direction of this misestimation.

So far, the two newly recommended statistical approaches, the Glejser test and quadratic regression, have been applied alongside the classical quartile test in the context of general intelligence^33^, intelligence facets^36^ as well as financial literacy^35^. The results were largely consistent across these domains: The classical, quartile analysis generally supported the Dunning–Kruger effect, the Glejser test gave mixed results, and the quadratic regression, which seems the most direct approach to examine the “unskilled but unaware” effect^35^, only gave tentative support for the effect in one of these domains (verbal intelligence^36^).

### Explorations of the Dunning Kruger effect in creative performance

The accuracy of self-assessments of creativity has been a topic of interest for some time^37,38^. However, according to our best knowledge, there have only been two studies that specifically examined the Dunning–Kruger effect in creativity. The first one used the classical quartile method^39^ and found that, across the whole sample people overestimated their creative performance in three different creative thinking tasks (the Similarities Test, the Remote Associates Test, and the Product Improvement Task). However, when looking at the quartiles separately, the classical Dunning–Kruger pattern was observed: Those in the highest quartile tended to underestimate their creativity, while those in lower quartiles had a tendency towards self-inflated appraisals. Interestingly, the overestimation of the lower quartile was smaller compared to classical studies^20^.

The second study examined the Dunning Kruger effect in divergent thinking (assessed with the Alternate Uses Test) across four educational stages (preschool, elementary school, high school, and undergraduate^40^). Taking into account the problems with the classical data analysis, the authors computed a non-hierarchical cluster analysis and identified three separate clusters: an overestimating group of unskilled and unaware participants (27.1%), an underestimating group of skilled and unaware participants (44.3%), and, most surprisingly, a group of unskilled but aware people (28.6%). The unskilled and unaware participants were mostly in the group of preschoolers, while the most skilled but unaware were among the undergraduate students, suggesting that effects were moderated by age groups. In sum, this methodological approach overcame limitations of traditional quartile-based analysis and offers new insights into developmental increases of creative metacognition with age. Still, it remains unclear whether other recent methods support a Dunning–Kruger effect with respect to creative ideation performance in adults.

### Present study

The main aim of this work was to test whether there is a *Dunning–Kruger effect* in creativity, as assessed by divergent thinking performance, and whether observation of this effect depends on the employed statistical methods including the classical quartile analysis as well as the Glejser test of heteroscedasticity and nonlinear (quadratic) regression. To this end, we conducted two independent studies. In the first study, we expected to confirm the Dunning–Kruger effect when using the classical analysis^39^ but did not make predictions regarding the two recently recommended approaches. In the second, preregistered study, we tested the robustness of our findings by examining if results of first study replicate.

For a more thorough analysis of the conditions of potential Dunning–Kruger effects, we further explored whether it plays a role how and when people make their self-assessments. Absolute self-assessments (I’m good at XY) and relative self-assessments (I’m better than others in XY) are crucial for gaining a deeper understanding of measurement by participants and more important thought to be distinct in nature^3,41–43^, so we included both. Kruger and Dunning^20^ proposed that high performers assume that others' skills are similar to their own, so they may underestimate themselves when using a relative scale, but not when making absolute estimates. On the other hand, low performers are likely to overestimate themselves in both cases^44^. Furthermore, we decided to ask participants to assess their performance before and after the tasks. It can be assumed that post-task assessments would be more accurate as the task itself provides valuable feedback^14,24, 45, 46^. Nevertheless, available meta-analytic findings showed little to no difference in accuracy based on whether self-assessments were made before or after performance^6,47–49^. By incorporating relative and absolute assessments, pre- and post-task assessments and applying both classical and recent statistical approach to test the Dunning–Kruger effect, we hoped to realize a thorough test of Dunning–Kruger effects in creativity, and, more generally, gain deeper insights into the accuracy of creative metacognition.

## Method

We conducted two online studies assessing divergent thinking (DT) ability with the established alternate uses task^50^ and asking participants to estimate their performance before and after the task. Given the similar methods, we describe both studies together in the following. Note, however, that both studies were independent, and the second study was preregistered ﻿(https://aspredicted.org/ey3bv.pdf) and realized only after completion of the first study.

### Sample

Both studies excluded data from participants who either a) missed any of the attention checks (i.e., two questions intermixed with the other questionnaire items), b) completed the study overly fast (< 1400 s), or c) did not generate at least one response to each DT task. Study 1 had a final sample of 425 participants (68.5% female, 31.1% male, 0.5% other), two-thirds of whom were University students (61.9%), and with a mean age of 29 years (*M* = 28.68, *SD* = 11.29, range: 18–69 years). In study 2, the final sample consisted of 317 participants (66.2% female, 33.1% male, 0.6% other) 59% of which were University students, and with a mean age of 30 years (*M* = 29.81, *SD* = 13.13, range = 17–73 years). According to Gignac and Zajenkowski (2020), 200 is the minimum sample size to be able to interpret quadratic regression analyses and Glejser correlations. For the classical analysis, our sample size ensures at least 80% power to detect within-quartile effects of *d* ≥  ± 0.4 (based on the smallest quartile with *n* = 73).

### Procedure

Participants completed all measures online via LimeSurvey. They first read and confirmed the informed consent form. In study 1 (conducted November–December 2021) and study 2 (conducted November–December 2022), divergent thinking tasks were completed. Before and after the tasks, participants provided self-assessments of their creativity. As these studies were part of a larger research project, participants completed additional tasks that were not in the focus of this work and thus are not reported here. The study was conducted according to the guidelines of the Declaration of Helsinki and the procedure had been approved by the ethics committee of University of Graz (GZ. 39/146/63 ex 2020/21).

### Materials

The data, code, and supplementary material with additional analysis are available on the OSF (https://osf.io/3kud6/).

### Measures

#### Creativity

Creative thinking performance was measured with divergent thinking tasks, precisely alternate uses tasks (AUT) which ask to generate creative uses for everyday objects^50^. In study 1, participants completed four AUT tasks (DT items were: brick, car tire, pen, can), two of which (randomly either the first or last two) were performed under “be creative” and “be fluent” instructions (i.e., asking to focus on the creative quality or quantity of responses;^51^, respectively.) For the assessment of DT creativity, we focused on the two items with “be creative” instructions. Five independent raters rated all responses on a scale ranging from zero (not creative at all) to four (very creative). Inter-rater reliability was high for all items, ICC between 0.82 and 0.83; internal consistency was decent for just two items with Cronbach’s alpha = 0.53. In study 2, participants performed three AUT tasks (DT items were: brick, car tire, pen) under “be creative” instructions and six independent raters rated all responses on the same scale. Inter-rater reliability was high for all tasks, ICC between 0.80 and 0.83; internal consistency across the three items was satisfactory with Cronbach’s alpha = 0.69. In both studies, we computed the max-3 score of DT creativity (i.e., the average of the three most creative responses according to average ratings) to address the potential confound between the creativity and fluency of responses^17,52^. As fluency (i.e., the number of generated uses) is another measure often included in creativity research but less central to the research questions at hand, we additionally report examinations of Dunning–Kruger effects for self-assessed versus actual fluency (under the “be fluent” condition; study 1) in the appendix (https://osf.io/3kud6/).

#### Self-assessed creativity

Before the DT tasks, participants indicated once how well they expect to perform in the task in general (absolute pre-task self-assessment) by reporting their agreement to the statement “I can come up with creative ideas” on a slider scale from 0 (do not agree at all) to 100 (totally agree). Next, they indicated how well they expect to do compared to others (relative pre-task self-assessment) on a slider scale from 0% (everybody else will have more creative ideas than me) to 100% (I will have more creative ideas than everybody else). After completing all DT tasks, participants responded to analogous questions in the past tense (absolute and relative post-task self-assessment).

#### Statistical analyses

We tested Dunning–Kruger effects under different conditions, including for (1) pre- and post-task self-assessments of creative performance, (2) absolute and relative self-assessments, and (3) using three different statistical approaches. The first test of the Dunning–Kruger effect was based on the classical method used by the original authors^20^. In line with them, we transformed our performance measures into percentile ranks to be able to compare them directly with the self-assessments (range: 0–100). In case of ties, we assigned each tied element the average rank (i.e., all participants with the same raw score also have the same rank). We then split our samples into quartiles based on their performance and compared self-assessments and performance (within-subjects factor *measure*) for the quartiles (between-subjects factor *quartile*) in ANOVAs^20^. We interpreted results as supporting a Dunning–Kruger effect if the ANOVA resulted in a significant interaction and the pairwise comparisons indicated that the lowest quartile showed the largest positive difference between self-assessment and performance (overestimation).

As a second way of testing the Dunning–Kruger effect, we computed the Glejser correlation^34^, a measure of the heteroscedasticity of residuals^33^. To calculate Glejser correlations, we (1) conducted linear regressions predicting self-assessed creativity from objectively measured creativity, (2) transformed the resulting residuals into absolute values, and (3) correlated these absolute residuals with objectively measured creativity. Here, a significantly negative correlation would be indicative of a Dunning–Kruger effect.

As a third test of the Dunning–Kruger effect, we conducted quadratic regressions, which Gignac^35^ argued to be less ambiguous than the Glejser correlation. In hierarchical regressions, we first entered a linear performance term into a model explaining self-assessment and then added a quadratic performance term. Following the recommendations by Gignac and Zajenkowski^33^, we considered these analyses as supporting a Dunning–Kruger effect if the Δ*R*^2^ between step one and two as well as the *sr*^2^ of the quadratic term were significant. Of note, since the quadratic term is the only predictor entered in the second step of these analyses, its sr^2^ is identical to the ΔR^2^ between step one and two. For this reason, we only report ΔR^2^.

As neither the Glejser test nor quadratic regression require a direct numerical comparison of self-assessment and performance, we conducted these analyses based on untransformed data (rather than percentiles) to preserve a maximum amount of information and higher scale of measurement^36^. As some readers might be interested in a direct comparison of the results of our classical and alternative analyses, we provide percentile-based results on the latter in the appendix. In short, these additional analyses showed virtually identical results in the Glejser tests and yielded no support for Dunning–Kruger effects in quadratic regressions. To counter potential violations of distributional assumptions, we based our interpretations on 95% bootstrapped confidence intervals based on 2000 samples.

## Results

### Descriptive statistics and intercorrelations

Table 1 contains the descriptive statistics and intercorrelations of all main variables for both studies. In both samples, the different creativity self-assessment measures were highly correlated with each other. Correlations between self-assessments and measured creativity were small too moderate, being descriptively somewhat higher in post-task assessments compared to pre-task assessments. We further report effect sizes (*d*) for statistical comparisons between mean self-assessed and measured creativity percentile (i.e., absolute accuracy). Interestingly, average differences between self-assessments and performance were only minor and, in most cases, (especially for relative self-assessments compared to others, i.e., relative) negative, meaning that people tended to underestimate themselves slightly.

**Table 1:** Descriptive Statistics, Differences between, and Intercorrelations of Self-Assessed and Measured Creativity (Study 1).

Study		Variable	Min–Max	*M* (*SD*)	*d*	1	2	3	4	5
1	1	Absolute Pre-SA	0.00–100.00	51.20 (20.29)	0.03					
	2	Relative Pre-SA	0.00–100.00	45.16 (18.19)	− 0.16*	0.82				
	3	Absolute Post-SA	0.00–100.00	46.61 (20.92)	− 0.12*	0.64	0.59			
	4	Relative Post-SA	0.00–100.00	42.64 (19.36)	− 0.25*	0.62	0.73	0.85		
	5	DT (raw)	0.17–2.92	1.88 (0.40)		0.20	0.15	0.31	0.26	
	6	DT (%)	0.24–100.00	50.12 (28.90)		0.24	0.20	0.32	0.28	0.95
2	1	Absolute Pre-SA	5.00–100.00	54.29 (21.28)	0.12*					
	2	Relative Pre-SA	0.00–100.00	46.11 (19.25)	− 0.13*	0.75				
	3	Absolute Post-SA	0.00–100.00	50.91 (20.60)	0.02	0.48	0.50			
	4	Relative Post-SA	0.00–100.00	45.22 (19.35)	− 0.16*	0.53	0.67	0.81		
	5	DT (raw)	0.64–2.94	1.79 (0.39)		0.14	0.17	0.23	0.26	
	6	DT (%)	0.32–100.00	50.16 (28.91)		0.15	0.17	0.25	0.28	0.97

Measured divergent thinking (DT) creativity is given as raw and percentile values (%); SA = Self-assessed creative performance; Absolute/Relative = Self-assessment in general/compared to others; Pre/Post = Self-assessments pre/post DT task performance. *d*s are Cohen’s *d*s for the differences between self-assessment and performance percentile (positive values = overestimation) with * indicating significance in two-tailed *t*-tests (*p* < *.*05). At *n* = 425 (study 1), all *r* ≥ 0.09 are significant at *p* < 0*.*05 and all *r* ≥ 0.16 are significant at *p* < 0*.*001. At *n* = 317 (study 2), all *r* ≥ 0.11 are significant at *p* < 0*.*05 and all *r* ≥ 0.18 are significant at *p* < 0*.*001.

### Dunning–Kruger effects

#### Classical analyses

**Figure 1:**
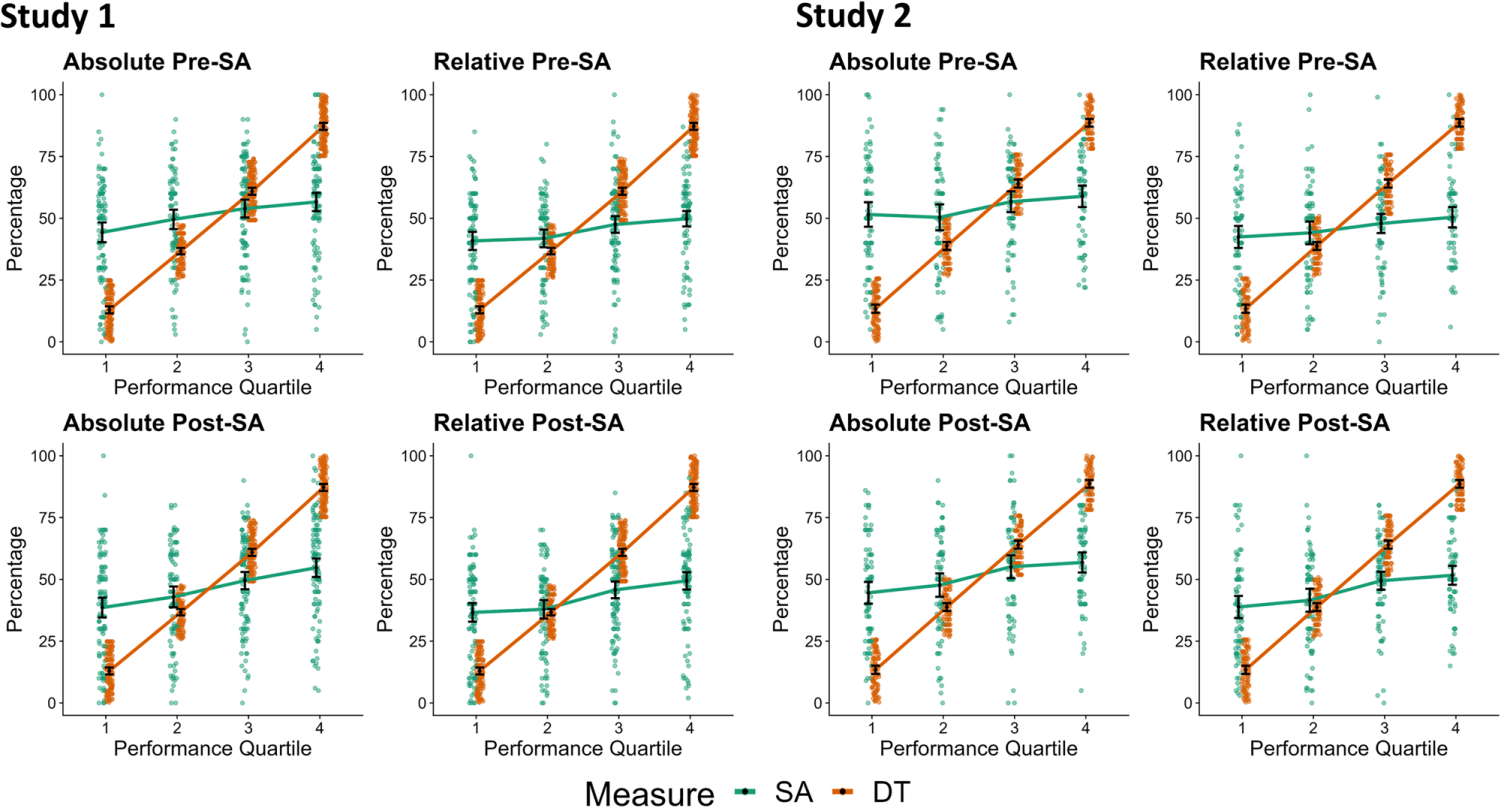
Quartile-based Tests: Self-Assessed and Measured Creativity for DT Creativity Quartiles. Colored dots indicate jittered participant-level data; black dots with error bars indicate means with 95% confidence intervals. *DT* divergent thinking performance. *SA* self-assessment.

Classical analyses showed support for Dunning–Kruger effects in all four conditions and across both studies. The relevant interaction between *measure* and *quartile* was significant in all ANOVAs (see Table 2). Pairwise comparisons also showed a pattern indicative of a Dunning–Kruger effects (see Table 3 and Fig. 1): People in the lowest quartile overestimated themselves the most. In five out of eight analyses, the second quartile also showed significant albeit considerably lower overestimation. Those in the highest quartile—and to a lesser degree also those in the second-to-highest quartile—were prone to underestimate themselves.

**Table 2:** Quartile-based tests: main effects and interactions of 2 (measure: self-assessed vs. measured creativity) × 4 (Creativity quartile) analyses of variance.

Study	SA condition	Effect	*F*	*DF*_n_	*DF*_d_	*p*	η^2^_g_
1	Absolute Pre-SA	Quartile	323.16	3	421	< 0.001	0.547
		Measure	2.73	1	421	0.099	0.003
		Quartile × Measure	181.04	3	421	< 0.001	0.381
	Relative Pre-SA	Quartile	378.92	3	421	< 0.001	0.575
		Measure	22.47	1	421	< 0.001	0.026
		Quartile × Measure	225.63	3	421	< 0.001	0.445
	Absolute Post-SA	Quartile	330.79	3	421	< 0.001	0.564
		Measure	9.33	1	421	0.002	0.010
		Quartile × Measure	162.95	3	421	< 0.001	0.344
	Relative Post-SA	Quartile	363.58	3	421	< 0.001	0.580
		Measure	55.58	1	421	< 0.001	0.058
		Quartile × Measure	196.05	3	421	< 0.001	0.395
2	Absolute Pre-SA	Quartile	200.40	3	313	< 0.001	0.496
		Measure	6.44	1	313	0.012	0.010
		Quartile × Measure	134.59	3	313	< 0.001	0.386
	Relative Pre-SA	Quartile	234.55	3	313	< 0.001	0.539
		Measure	19.78	1	313	< 0.001	0.029
		Quartile × Measure	163.01	3	313	< 0.001	0.429
	Absolute Post-SA	Quartile	241.00	3	313	< 0.001	0.547
		Measure	0.02	1	313	0.884	0.000
		Quartile × Measure	128.66	3	313	< 0.001	0.371
	Relative Post-SA	Quartile	274.98	3	313	< 0.001	0.580
		Measure	28.28	1	313	< 0.001	0.041
		Quartile × Measure	143.50	3	313	< 0.001	0.395

SA = Self-assessed creative performance; Absolute/Relative = Self-assessment in general/compared to others; Pre/Post = Self-assessments pre/post DT task performance.

η^2^_g_ = generalized eta square. *n*_Study 1_ = 425. *n*_Study 2_ = 317.

**Table 3:** Post-tests for quartile-based tests: pairwise comparisons of self-assessed versus measured creativity (%) per DT creativity quartile.

Study	SA condition	Quartile	*t*	*df*	*M*_diff_	95% BCa *CI*	*p*	*d*
1	Absolute Pre-SA	1	14.15	108	31.36	[27.08; 35.41]	< 0.001*	1.36
		2	6.53	92	12.85	[8.87; 16.63]	< 0.001*	0.68
		3	− 3.93	112	− 7.01	[− 10.48; − 3.57]	< 0.001*	− 0.37
		4	− 15.39	109	− 30.58	[− 34.43; − 26.91]	< 0.001*	− 1.47
	Relative Pre-SA	1	13.57	108	27.90	[23.86; 31.83]	< 0.001*	1.30
		2	2.75	92	5.15	[1.53; 8.70]	0.007*	0.29
		3	− 7.54	112	− 13.45	[− 16.79; − 9.83]	< 0.001*	− 0.71
		4	− 21.57	109	− 37.36	[− 40.75; − 34.10]	< 0.001*	− 2.06
	Absolute Post-SA	1	12.62	108	25.65	[21.56; 29.61]	< 0.001*	1.21
		2	2.96	92	6.21	[2.23; 10.29]	0.002*	0.31
		3	− 6.44	112	− 11.44	[− 15.01; − 8.02]	< 0.001*	− 0.61
		4	− 16.43	109	− 32.46	[− 36.34; − 28.63]	< 0.001*	− 1.57
	Relative Post-SA	1	12.25	108	23.70	[20.12; 27.47]	< 0.001*	1.17
		2	0.61	92	1.17	[− 2.45; 4.86]	0.545	0.06
		3	− 8.55	112	− 15.18	[− 18.83; − 11.79]	< 0.001*	− 0.80
		4	− 19.92	109	− 37.79	[− 41.58; − 34.19]	< 0.001*	− 1.90
2	Absolute Pre-SA	1	14.43	83	38.12	[32.90; 43.26]	< 0.001*	1.57
		2	4.22	76	11.58	[6.23; 16.92]	< 0.001*	0.48
		3	− 3.31	82	− 7.34	[− 11.31; − 2.85]	< 0.001*	− 0.36
		4	− 13.40	72	− 29.77	[− 34.11; − 25.58]	< 0.001*	− 1.57
	Relative Pre-SA	1	12.54	83	29.03	[24.48; 33.47]	< 0.001*	1.37
		2	2.20	76	5.30	[0.70; 10.07]	0.034	0.25
		3	− 7.63	82	− 16.15	[− 20.47; − 12.18]	< 0.001*	− 0.84
		4	− 18.02	72	− 38.23	[− 42.45; − 34.09]	< 0.001*	− 2.11
	Absolute Post-SA	1	13.10	83	31.16	[26.45; 35.68]	< 0.001*	1.43
		2	3.61	76	8.89	[3.83; 13.72]	< 0.001*	0.41
		3	− 3.76	82	− 8.93	[− 13.53; − 4.46]	< 0.001*	− 0.41
		4	− 15.27	72	− 31.81	[− 35.88; − 27.61]	< 0.001*	− 1.79
	Relative Post-SA	1	10.92	83	25.43	[20.99; 29.99]	< 0.001*	1.19
		2	1.12	76	2.74	[− 2.07; 7.46]	0.261	0.13
		3	− 7.22	82	− 14.58	[− 18.60; − 10.70]	< 0.001*	− 0.79
		4	− 19.26	72	− 37.00	[− 40.74; − 33.33]	< 0.001*	− 2.25

SA Crea = Self-assessed creative performance; Absolute/Relative = Self-assessment in general/compared to others; Pre/Post = Self-assessments assessed pre/post DT task performance. *n*_Study 1_ = 425. *n*_Study 2_ = 317. * = significant after Bonferroni-correction (.05/4: *p* < 0.013). Confidence intervals are based on 2,000 bootstrap samples. Positive values for *t* and *d* indicate that measured creativity is higher than self-assessed creativity (i.e., overestimation).

#### Glejser test of heteroscedasticity

**Figure 2:**
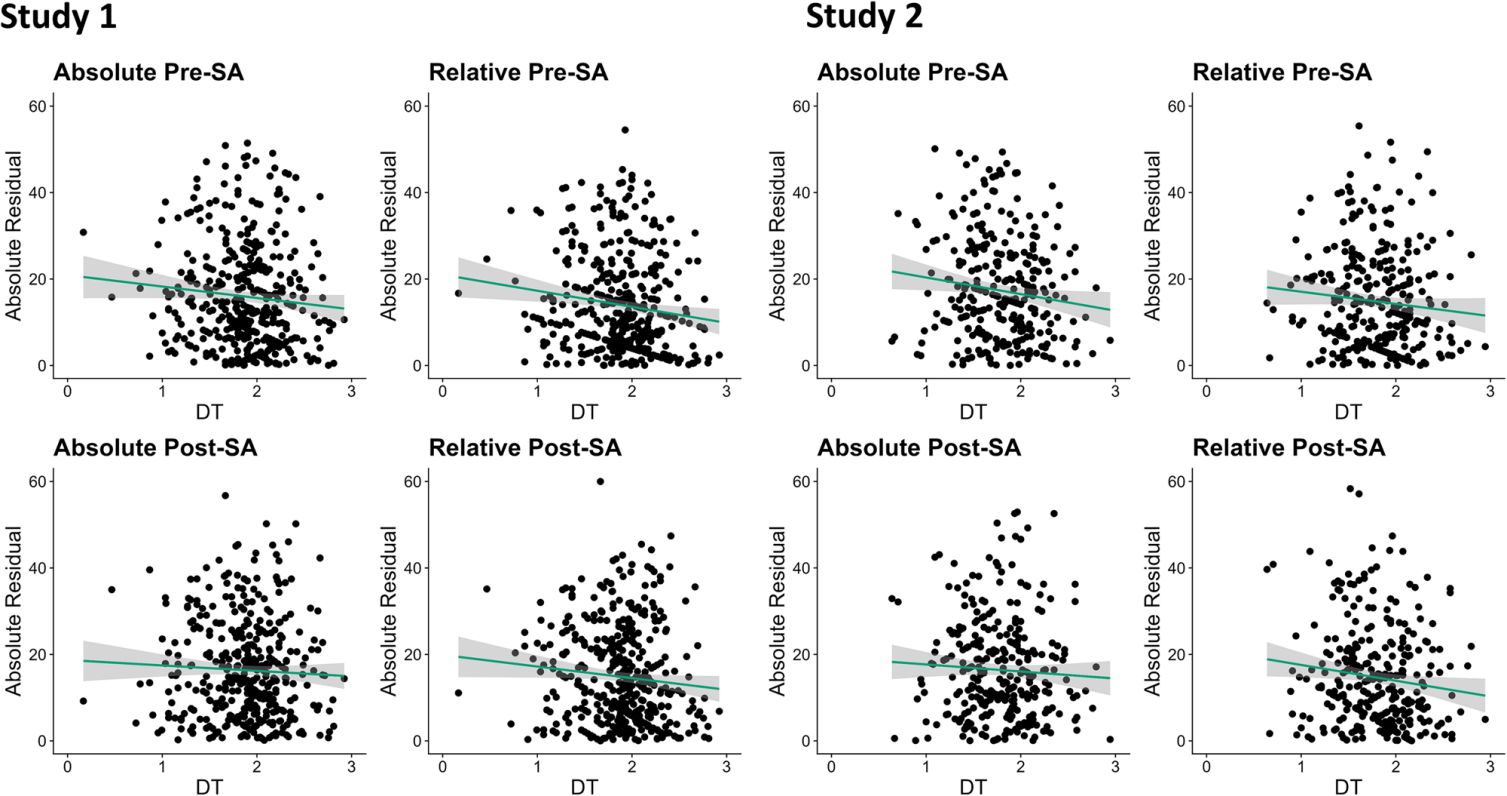
Glejser Correlations of Heteroscedasticity. Lines and shaded areas around them represent linear associations with 95% confidence bands. *DT* divergent thinking performance. *SA* self-assessment.

On a merely descriptive level, we observed small negative correlations between absolute residuals and DT performance across all conditions (see Fig. 2), but they reached statistical significance only in specific cases (see Table 4). In study 1, we found support for Dunning–Kruger effects in relative pre- and post-task self-assessments but not in absolute pre- or post-task self-assessments. While the Glejser correlation for relative post-task self-assessments was also significant in study 2, the one for relative pre-task self-assessments was not. Moreover, in study 2 absolute pre-task self-assessments also showed a significant negative Glejser correlation, while the respective correlation for absolute post-task self-assessments was still not significant. Thus, only one of four conditions showed consistent Dunning–Kruger effects in both samples (relative post-task self-assessment).

**Table 4:** The Glejser test of heteroscedasticity.

Study	SA condition	*r*	*CI*	*p*
1	Absolute Pre-SA	− 0.090	[− 0.178; 0.000]	0.050
	Relative Pre-SA	− 0.134	[− 0.219; − 0.045]	0.004
	Absolute Post-SA	− 0.045	[− 0.145; 0.048]	0.369
	Relative Post-SA	− 0.096	[− 0.187; − 0.004]	0.046
2	Absolute Pre-SA	− 0.126	[− 0.228; − 0.018]	0.015
	Relative Pre-SA	− 0.094	[− 0.189; 0.008]	0.072
	Absolute Post-SA	− 0.055	[− 0.166; 0.053]	0.309
	Relative Post-SA	− 0.124	[− 0.232; − 0.010]	0.032

SA = Self-assessed creative performance; Absolute/Relative = Self-assessment in general/compared to others; Pre/Post = Self-assessments assessed pre/post DT task performance. *n*_Study 1_ = 425. *n*_Study 2_ = 317. Values in brackets are 95% bias-corrected and accelerated confidence intervals based on 2000 bootstrap samples.

#### Non-linear regression

**Figure 3:**
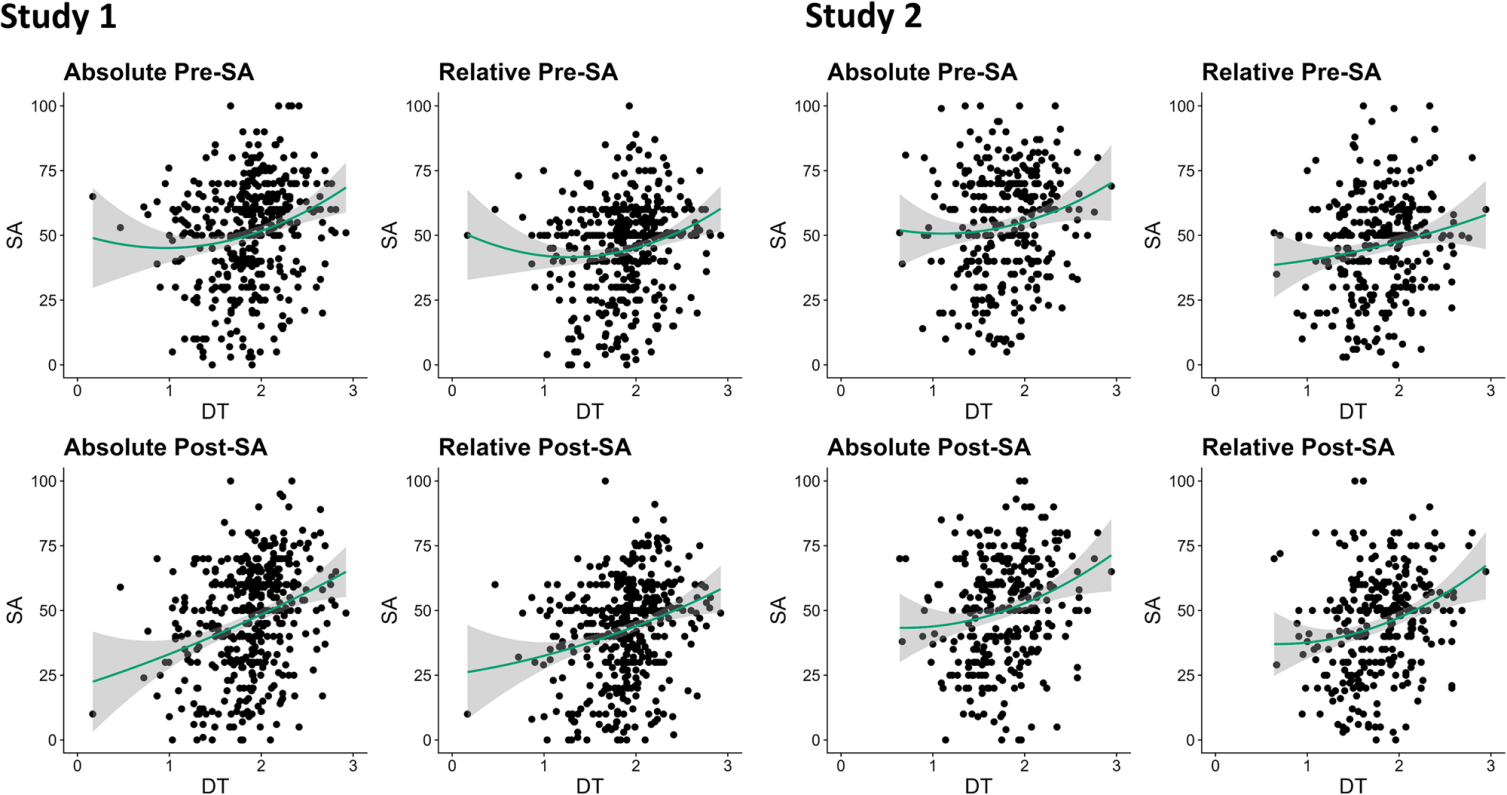
Nonlinear regression between Measured and Self-Assessed Creativity. Lines and shaded areas around them represent quadratic lines of best fit with 95% confidence bands. *DT* divergent thinking performance. *SA* Self-assessment.

Across both studies and all conditions, the first step of hierarchical multiple regressions indicated significant linear effects of creative performance on self-assessments (all *R*^2^ ≥ 0.02 & ≤ 0.10; all *p* ≤ 0.01, all *F* ≥ 6.51). However, only in one condition of study 1 (relative pre-task self-assessment) the quadratic effect of performance significantly contributed to the model in the second step (Δ*R*^2^ = 0.01, 95% CI [> 0.00, 0.03], *F*(1*,* 422) = 4*.*49, *p* = 0.035; all other *p* ≥ 0.096, all other *F* ≤ 2.79). Thus, these analyses only support a Dunning–Kruger effect for self-assessments made before the task in comparison to others (see also Fig. 3). Notably, this finding did not replicate in study 2 (Δ*R*^2^ < 0.001, 95% CI [0.00, 0.01], *F*(1*,* 314) = 0*.*15, *p* = 0.702).

## Discussion

Creative challenges are often ill-defined, with unclear evaluation criteria and infinite possible solutions. Therefore, self-assessment may be vital in regulating the creative process. At the same time, these specifics of creative tasks could make self-assessments in this area particularly hard, potentially leading to lower accuracy and higher biases^11–13^. Past research indicated that creative people are doubly skilled in not only generating more creative ideas but also being able to judge the creativity of ideas more accurately^10,17–19^. Still, the question remains whether creative people are also more accurate in judging their creative performance overall, that is, if they have higher creative metacognitive monitoring skills at the performance-level, not just at the response-level (idea evaluation)^14^. Or put differently, do less creative people judge their creative performance less accurately, and hence, does the Dunning–Kruger effect extend to creativity? As available findings are not fully conclusive^39,40^ and may partly depend on the employed test approach^35^, this work aimed for a comprehensive test of Dunning–Kruger in creative ideation performance with different methods.

Contrary to the assumption that self-assessments in the context of creativity might be particularly challenging, we found that metacognitive monitoring accuracy at performance level aligned well with past results from other domains^5,6^ with correlations between self-assessment and creative performance ranging between 0.1 and 0.3 (see Table 1). Of note, these correlations are still low enough to question people’s self-insight when it comes to creative performance. At least descriptively, creative self-assessments made after the task were slightly more accurate than those made before it. Thus, gaining experience with the task might help people calibrate their self-assessments to their performance, which also has been previously shown, although not consistently^24,45, 46^, but see^6,47–49^.

When analyzing differences between self-assessments and performance, we found small but statistically significant underestimation effects for the majority of conditions. This result is surprising, considering previous findings in creativity research^39^ and the general tendency of people to judge themselves as above average^29,30^. But it is worth noting that a small number of studies also reported underestimation of performance in other areas like intelligence^24,36, 53^. In fact, creative people tend to underestimate the originality of their ideas^16^, which may in consequence also let them underestimate their overall performance^54^. Notably, self-assessments were consistently below average when people compared themselves to others, whereas self-assessments were sometimes above average when people assessed their creative performance in general (relative versus absolute self-assessment). This pattern suggests that self-assessments are specific to the type of question asked, allowing people to view their performance as creative, yet potentially less creative compared to others^40^. In sum, we observed a below-average effect when people compare their creative performance to others, which may be due to inherent uncertainty of creative processes (compared to other domains where people know when they solved a task correctly) which eventually decreases people's confidence.

Our main research question was if uncreative people are particularly inaccurate in their performance assessment as assumed by the Dunning–Kruger effect. When we considered the classical quartile analysis employed by the original authors^20^, we found consistent support for the Dunning–Kruger effect across two samples and four self-assessment measures^39^: The lowest quartile overestimated themselves the most. Additionally, the classical analyses revealed significant underestimation in the highest performance quartile across all conditions and not just for relative self-assessments. This latter finding stands in contrast with the assumption that the negative bias of high performers is mainly due to the false consensus effect (i.e., overestimation of the others instead of underestimation of oneself^20^). Notably, underestimation effects of high-performers were descriptively even higher for relative self-assessments (*d*s between − 1.92 and − 2.25) compared to absolute self-assessments (*d*s between − 1.55 and − 1.79). This could mean that high performers both underestimate themselves and overestimate others. Still, overestimation for low performers and underestimation for high performers could also be simply due to regression to mean effects. Therefore, we used additional statistical tests that are potentially less affected by regression-to-the mean effects.

In line with research from other domains^33,35, 36^, the consistent Dunning–Kruger effects observed in the classical analyses stood in stark contrast to the results obtained when employing the statistical alternatives suggested by Gignac and Zajenkowski^33^. When we applied the Glejser test of heteroscedasticity^34^, only relative self-assessments made after the task showed a consistently negative Glejser correlation across both samples. It can be conceded that all eight Glejser correlations were negative at a descriptive level. This may point to a small but consistent effect of less creative people being minimally less accurate to be established in more powerful analyses (n > 780 is needed to establish *r* = 0.1 with a power of 0.80), albeit with little practical significance. The support for the Dunning–Kruger effect was even weaker in quadratic regression analyses: Only one result was in favor of the Dunning–Kruger effect (relative self-assessments made before the task in study 1), but this was not replicated in study 2, suggesting that self-assessments and performance do not correlate consistently higher in more creative people. Of note, the results of our secondary analyses based on DT fluency of AUT responses (see appendix) were mostly in line with findings on DT creativity. In sum, our work adds to the growing body of literature finding only limited support for the Dunning–Kruger effect beyond regression to the mean effects^23,26^.

This study has several strengths, including the use of two large samples, relative and absolute assessments, pre- and post-task assessments, and classical and recent statistical approaches which enabled a comprehensive test of the Dunning–Kruger effect in the context of creative performance. However, it is essential to consider some limitations when interpreting the results. First, the samples involved a large proportion of university students, so caution should be taken when generalizing the results to other groups—maybe creative professionals can judge their creativity more accurately. Second, the data was collected online, which implies lower experimental control compared to lab experiments, but also guaranteed highest anonymity which could support honest self-assessments. Third, we assessed creative performance only in the context of divergent thinking ability as assessed with the AUT. While this is arguably the most dominant approach for assessing creative ability in creativity research^55^, the Dunning–Kruger effect could further be explored with other, more complex creativity tasks, and potentially domain-specific creativity measures. Future research thus may aim to replicate our findings in a more diverse sample and controlled context but also look beyond performance measures to predict the creative metacognitive monitoring accuracy and consider other relevant variables like personality or previous experiences with tasks. We further need to acknowledge that the reliability of DT assessments was modest and that this was likely due to the low number of DT items. While using 2–3 items for DT assessment is common practice^56^, it may not be enough to ensure highly reliable DT creativity measures—an issue that was not apparent to us as many previous studies (76%) failed to report the reliability of their DT assessments^56^. It is well known that (low) reliability limits the potential strength of correlations (in this case between DT and self-assessments) and past work showed that reliability tends to be even lower at the extremes of the distribution^57,58^. If associations between self-assessments and performance are lower at the extremes (i.e., in line with a DK effect), this might partly simply be a function of lower reliability^57^. It is unclear whether this effect is aggravated by an overall low reliability. However, future studies focused on DT, the DK effect, and particularly their interaction would benefit from paying closer attention to reliability.

In conclusion, this work found no strong support for a Dunning–Kruger effect in creativity. The systematic overestimation/underestimation of own performance in low versus high performers may be largely due to regression to mean effects. Interestingly, the creative domain may stand out in not being subject to common above-average biases but, if anything, rather exhibit a below-average effect. More generally, people appear to have only rather limited insight in their creative performance level—some overestimate, others underestimate themselves, and yet others seem to be good judges of their creative performance. This raises the question of what other factors might predict an accurate assessment of one’s own creativity if it is not creative performance itself. While being more creative supports discernment in judging the creativity of one’s own and others’ ideas, it may not come with higher accuracy in judging own creative task performance. So, creative people may only be doubly blessed, implying at least only a dual rather than a triple-burden for less creative people.

## Data Availability

The data, code, and supplementary material with additional analysis are available on the OSF (https://osf.io/3kud6/).
